# Neuromodulation to Promote Recovery Following Traumatic Brain Injury: A Narrative Review of Current Pharmacologic and Non-Pharmacologic Approaches

**DOI:** 10.3390/brainsci16080813

**Published:** 2026-07-30

**Authors:** Cindy K. Wong, Nilsha Khurana, Raya T. Aliakbar, Roy A. Poblete

**Affiliations:** 1 Department of Neurology, Keck School of Medicine, University of Southern California, Los Angeles, CA 90033, USA; cindy.wong2@med.usc.edu (C.K.W.); nilsha.khurana@med.usc.edu (N.K.); 2 Department of Neurology, Los Angeles General Medical Center, Los Angeles Department of Health Services, Los Angeles, CA 90033, USA; 3 Department of Neurology, Stanford Health Care, Stanford School of Medicine, Palo Alto, CA 94305, USA; rtalia@stanford.edu

**Keywords:** traumatic brain injury, neuromodulation, neurorehabilitation, disorders of consciousness

## Abstract

Traumatic brain injury (TBI) is a leading cause of long-term neurological disability worldwide and is frequently associated with persistent impairments in consciousness, cognition, mood, and functional independence. Despite advances in acute neurocritical care, effective therapies that enhance neurological recovery remain limited. Neuromodulation has emerged as a promising strategy to augment neuroplasticity and improve recovery through both pharmacologic and non-pharmacologic approaches. This narrative review summarizes current evidence supporting pharmacologic neuromodulatory therapies, including central nervous system stimulants (methylphenidate and modafinil), dopaminergic agents (amantadine and bromocriptine), acetylcholinesterase inhibitors (donepezil and rivastigmine), and selective serotonin reuptake inhibitors (sertraline and fluoxetine). Mechanisms of action, clinical efficacy, adverse effects, and practical considerations across the acute, subacute, and chronic phases of TBI recovery are discussed. Emerging non-pharmacologic neuromodulation techniques, including repetitive transcranial magnetic stimulation, transcranial direct current stimulation, electroconvulsive therapy, vagus nerve stimulation, and deep brain stimulation, are also reviewed. Although amantadine remains the only neuromodulator supported by moderate-quality guideline recommendations for accelerating recovery in disorders of consciousness, accumulating evidence suggests that several additional pharmacologic and non-pharmacologic neuromodulation interventions may improve attention, executive function, fatigue, mood, and rehabilitation participation in carefully selected patients. However, current evidence is limited by heterogeneous study populations, small sample sizes, inconsistent outcome measures, and a paucity of long-term randomized controlled trials. Future research should prioritize adequately powered comparative studies, standardized outcome measures, biomarker-guided patient selection, and multimodal treatment strategies to optimize neurological recovery following TBI.

## 1. Introduction

Traumatic brain injury (TBI) is a significant global health concern, affecting an estimated 69 million persons annually. The resulting public health burden emphasizes the need for targeted prevention and management strategies [1]. TBI survivors often endure a spectrum of disabilities that impair physical, cognitive, emotional, and social functioning, negatively impacting quality of life. Cognitive impairments are prevalent, with up to 62% experiencing deficits in memory, slowed processing, and difficulties with concentration [2]. In addition, 25% of severe TBI patients report anxiety, and 23.7% experience depression [3]. Physical disabilities are reported by 47.5% of patients, including balance and motor difficulties [3]. Given the morbidity, there is an urgent need to develop novel treatment modalities that promote recovery across multiple neurologic domains to enhance functional outcomes and quality of life.

At the biochemical level, moderate and severe TBI causes widespread neuronal damage in pathways critical for maintaining consciousness and cognitive function [4]. One key pathological mechanism is excitotoxicity, where excessive synaptic release of glutamate and decreased reuptake prolong neuronal receptor activation, leading to cell death or dysfunction [5,6]. Additionally, TBI induces a cascade of neurochemical changes, including inflammation, oxidative stress, and altered cerebral blood flow, that contribute to disorders of consciousness (DoCs) [6]. Despite a lack of high-quality evidence and guidelines, neuromodulators have been increasingly used in clinical practice to promote wakefulness and cognitive recovery after acquired brain injuries. Medications such as amantadine, methylphenidate, and modafinil have shown promise in improving attention, arousal, and motivation, thereby enhancing patient participation in rehabilitative therapies [7]. Nevertheless, the use of neuromodulators requires careful consideration of individual patient needs and potential side effects.

In parallel with pharmacologic therapies, non-pharmacologic neuromodulation has emerged as a promising adjunctive strategy to enhance neurological recovery after TBI. Techniques such as repetitive transcranial magnetic stimulation (rTMS), transcranial direct current stimulation (tDCS), vagus nerve stimulation (VNS), and deep brain stimulation (DBS) aim to modulate dysfunctional cortical and subcortical networks, promote activity-dependent neuroplasticity, and facilitate recovery of cognition, mood, arousal, and motor function [8,9,10,11,12,13]. Although these approaches remain investigational for many indications, early clinical evidence suggests they may augment conventional multidisciplinary rehabilitation and complement pharmacologic neuromodulation in carefully selected patients.

Several recent reviews have summarized individual neuromodulatory interventions for TBI. However, most have focused on a single therapeutic modality or specific symptom domain. In contrast, this review integrates both pharmacologic and non-pharmacologic neuromodulation strategies, incorporates current guideline recommendations, emphasizes phase-specific treatment considerations across the acute, subacute, and chronic stages of recovery, and discusses future directions for multimodal and precision-based neurorehabilitation.

This comprehensive narrative review assesses the current literature to provide clinicians and scientists with insights into the use of neuromodulators, including central nervous system (CNS) stimulants, dopaminergic agents, acetylcholinesterase (AChE) inhibitors, and selective serotonin reuptake inhibitors (SSRIs), to support neurological recovery in patients with TBI (Table 1). Additionally, we explore non-pharmacological and investigational approaches and propose future directions for translational research to improve outcomes in TBI rehabilitation. Throughout this review, we discuss the timing of neuromodulatory interventions whenever evidence is available, referring to the acute (<1 month), subacute (1–6 months), and chronic (>6 months) phases of TBI recovery.

## 2. Disorders of Consciousness Following TBI

TBI frequently results in DoCs, characterized by impairments in arousal, awareness, or cognitive functions such as attention and memory. Among moderate-to-severe TBI patients, approximately one-half present with impaired consciousness, with 10–15% remaining impaired at hospital discharge. In severe TBI, approximately one-third have persistent coma, vegetative state (also called unresponsive wakefulness syndrome), or minimally conscious state at three weeks, but significantly declining by three months [14,15,16]. DoCs are hypothesized to result from disruptions in key neuronal pathways that maintain consciousness. Specifically, the reticular activating system (RAS), located within the dorsal brainstem and extending into the midbrain and thalamus, regulates wakefulness and arousal [17]. It sustains cortical activation and interacts with neurotransmitter systems, such as acetylcholine (ACh) and norepinephrine (NE), to enhance alertness and attention. Thalamocortical networks also play a crucial role in processing sensory information to maintain alertness, and the basal forebrain supports these functions by releasing ACh [17]. Disruption of these pathways increases the risk of developing DoCs. Phenotypically, this can result in a coma or encephalopathy with a depressed level of consciousness.

The pathophysiology of DoC following TBI is complex. Following the primary mechanical insult, secondary injuries involve a cascade of neuroinflammation-mediated mechanisms. Activated microglia and astrocytes release pro-inflammatory cytokines, such as interleukin-1β (IL-1β), interleukin-6 (IL-6), and tumor necrosis factor-α (TNF-α) [18]. While these cytokines play a role in brain repair by clearing cellular debris and initiating remodeling and neuroplastic processes, their dysregulation leads to chronic inflammation that contributes to secondary brain injury. Concurrently, TBI triggers a metabolic crisis characterized by an imbalance between the brain’s energy demands and its capacity to supply that energy [19]. Mitochondrial dysfunction further impairs ATP production. This energy deficit is exacerbated by oxidative stress from the overproduction of reactive oxygen species (ROS) that overwhelms antioxidant defenses [20]. ROS, including superoxide and hydroxyl radicals, cause extensive cellular damage and exacerbate inflammation, leading to self-propagating neuronal injury [20]. TBI can also disrupt the hypothalamic-pituitary axis, resulting in endocrine and hormonal imbalances like growth hormone deficiency, adrenal insufficiency, and hypothyroidism, all of which impact recovery. Hormonal disturbances contribute to fatigue, cognitive impairments, and metabolic disturbances, and inhibit neuroplasticity [21].

Pharmacologic neuromodulators seek to restore disrupted dopaminergic, noradrenergic, serotonergic, and cholinergic neurotransmission while also modulating excitotoxicity and neuroinflammatory cascades. Complementary non-pharmacologic neuromodulation techniques such as rTMS, tDCS, VNS, and DBS aim to restore dysfunctional thalamocortical and corticocortical network activity through activity-dependent neuroplasticity. However, given the complex pathophysiology of DoCs, multimodal approaches are needed. Given the complex pathophysiology of DoCs, combining pharmacologic neuromodulation, non-pharmacologic neuromodulation, and multidisciplinary rehabilitation may provide the greatest opportunity to promote recovery of consciousness and higher-order cognitive function.

## 3. Pharmacotherapy in TBI

Neuromodulation with pharmacological therapies targets specific neurotransmitter systems to enhance cognitive function and mood following TBI. CNS stimulants, such as methylphenidate and modafinil, increase dopamine (DA) and NE by inhibiting their reuptake, promoting wakefulness and attention through dopamine transporter (DAT) activation [22]. Dopaminergic agents support arousal in complementary ways. Amantadine increases presynaptic DA release, blocks DA reuptake, and—through weak N-methyl-D-aspartate (NMDA) antagonism with modest norepinephrine-transporter (NET) inhibition—amplifies postsynaptic dopaminergic signaling. Acetylcholinesterase inhibitors (AChEIs), such as donepezil and rivastigmine, increase ACh levels to enhance cognitive function and wakefulness, and regulate the sleep–wake cycle [23]. SSRIs, including fluoxetine and sertraline, increase serotonin levels to improve mood and anxiety [24]. They also promote neuroplasticity and neurogenesis through dopaminergic effects, thereby strengthening cognitive resilience and enhancing wakefulness [24]. The evidence supporting the use of each pharmacologic class will be discussed separately below.

Therapeutic efficacy and the strength of evidence for neuromodulatory therapies vary considerably across the acute, subacute, and chronic phases of TBI recovery [7,25,26,27,28]. Therefore, the timing of intervention should be considered when selecting pharmacologic therapy, as individual agents have demonstrated benefit during different stages of recovery. In the acute phase, neuromodulators can promote overt consciousness and arousal and facilitate engagement in rehabilitative therapies [25]. In the subacute and chronic phases, therapies can be tailored to the evolving needs of TBI survivors as they progress through recovery [26]. Studies have shown that neuromodulators can help sustain cognitive gains, improve mood and attention, and alleviate fatigue [7,27,28]. However, while neuromodulators hold promise across all stages of recovery, greater understanding is needed to refine treatment protocols, optimize dosing, and assess long-term efficacy after TBI. Future treatment paradigms will likely integrate pharmacologic and non-pharmacologic neuromodulation rather than relying on single interventions, with therapies tailored to the patient’s stage of recovery and clinical phenotype.

## 4. CNS Stimulants

### 4.1. Methylphenidate

Methylphenidate, a CNS stimulant widely prescribed for attention-deficit/hyperactivity disorder (ADHD) and narcolepsy, has drawn interest in TBI as a treatment for executive dysfunction and attentional deficits. Its primary mechanism of action involves inhibition of the DAT and NET, leading to elevated concentrations of DA and NE in the synaptic cleft [29]. This enhances neural activity in the striatum, nucleus accumbens, and prefrontal cortex—circuits responsible for attention, processing speed, and executive function [30]. Methylphenidate also increases DA availability by modulating vesicular monoamine transporter 2 (VMAT-2), supporting arousal and reducing mental fatigue [31,32].

Several small clinical studies suggest potential cognitive benefits. In a double-blind, placebo-controlled trial, Whyte et al. found that methylphenidate significantly improved processing speed, task-related attentiveness, and caregiver-rated attention as measured by a multidimensional attention battery [26]. Similarly, Al-Adawi et al. reported improvements in executive function on Digit Span and Verbal Fluency tests, particularly among patients with chronic TBI. Notably, benefits persisted after discontinuation of the drug, suggesting lasting therapeutic effects [33]. Peattie et al. used functional MRI during a Tower of London task to examine the neural mechanisms underlying treatment effects. In their randomized controlled trial (RCT), methylphenidate improved reaction time and visuospatial planning accuracy, and was associated with normalized activation in the bilateral inferior frontal gyri and insula [34]. With methylphenidate use, brain activation patterns more closely resembled those of healthy controls compared to TBI patients receiving a placebo. Connectivity across a broader visuospatial network also increased, highlighting methylphenidate’s impact on underlying neural circuitry [34].

A systematic review also supports the use of methylphenidate. A review conducted by Barnett and Reid concluded that the drug consistently improves executive functioning, including planning, organization, and cognitive flexibility, as well as processing speed, with benefits extending across several standardized neuropsychological measures [35]. Similarly, a meta-analysis by van der Veen et al. demonstrated positive effects on executive memory, baseline speed, and inhibitory control [36]. Although modest in magnitude, the consistency of these effects across multiple trials suggests a potential role for methylphenidate in cognitive rehabilitation. Limited long-term follow-up studies suggest sustained improvements in selected cognitive and neuropsychiatric outcomes, although additional high-quality studies are needed. In a 5-year follow-up study of patients with mild TBI, Johansson et al. reported sustained improvements in processing speed alongside reductions in mental fatigue, depression, and anxiety [37]. Most published studies have evaluated methylphenidate during the subacute and chronic phases of TBI recovery, where improvements in attention, processing speed, and executive function have been observed. Evidence supporting routine initiation during the acute neurocritical care period remains limited [26,35,36,37].

### 4.2. Modafinil

Modafinil has been investigated as a treatment for several post-TBI symptoms, most notably excessive daytime sleepiness (EDS), cognitive slowing, and DoC. Clinically, it is generally well tolerated and carries a favorable safety profile [38,39]. Its mechanism of action is not fully understood, but it is thought to increase DA levels by inhibiting DAT [39]. Unlike amphetamines, modafinil modulates a broader range of neurotransmitter systems, including serotonin, NE, histamine, and hypocretin, while enhancing glutamatergic activity and suppressing GABAergic tone [39]. It also engages hypothalamic arousal centers, such as the tuberomammillary nucleus and orexin neurons, mechanisms that align with its established use in narcolepsy, obstructive sleep apnea, and shift work disorder [40,41].

In TBI, evidence is most extensive in the treatment of EDS, a syndrome that disrupts daily functioning and limits participation in rehabilitation. An RCT by Kaiser et al. showed that modafinil was associated with significant reductions in EDS compared to placebo [42]. Observed improvements in alertness may be clinically meaningful, as better wakefulness supports engagement in therapy. Other studies have explored its effect on DoC. In a retrospective case series, Dhamapurkar et al. found that 11 of 12 TBI patients with prolonged DoC who were on modafinil demonstrated improved wakefulness, awareness, concentration, and command-following, as reflected in higher Wessex Head Injury Matrix scores [43]. A systematic review by Seifi et al. also suggested that modafinil was associated with improvements in Glasgow Coma Scale (GCS) scores [44]. Results, however, have been mixed. Borghol et al. reported no short-term improvements in GCS within 72 h of modafinil initiation. However, patients were observed to maintain high levels of participation in physical and occupational therapy—hinting that the drug may promote stamina and engagement even when immediate neurological change is not detectable [45]. Its direct effects on cognition and recovery of consciousness remain uncertain, highlighting the need for larger clinical trials that incorporate both neuropsychological and functional outcomes. Available evidence spans both acute and chronic TBI populations, although benefits are most consistently observed in patients with persistent fatigue or excessive daytime sleepiness during rehabilitation [42,43,44,45].

## 5. Dopaminergic Agents

### 5.1. Amantadine

In current clinical practice, amantadine is widely used in TBI, particularly in patients with DoC during the acute post-injury period. Although initially developed as an antiviral agent, it was later repurposed for the treatment of Parkinson’s Disease due to its dopaminergic properties. Its only FDA-approved neurological indications remain Parkinson’s Disease and drug-induced extrapyramidal symptoms, yet off-label use in TBI has become increasingly common as clinical evidence has emerged [46,47,48,49]. Amantadine’s therapeutic appeal lies in its pleiotropic mechanism of action. It functions as a weak, non-competitive NMDA receptor antagonist, moderating glutamatergic transmission and thereby reducing excitotoxic injury [48]. In parallel, it also delays DA reuptake and increases postsynaptic DA receptor expression, enhancing DA effects on arousal, attention, and motor recovery [49]. Additionally, inhibition of inward-rectifying potassium (Kir2) channels has been described, which may stabilize neuronal resting membrane potentials and support synaptic excitability during recovery [50].

The most significant clinical evidence was reported in a multicenter, randomized, double-blind, placebo-controlled trial by Giacino et al., which enrolled 184 patients in vegetative or minimally conscious states between four and sixteen weeks post-injury [47]. Over a four-week treatment period, those receiving amantadine demonstrated significantly faster functional recovery compared to placebo, as measured by Disability Rating Scale (DRS). Improvements occurred across domains of arousal, awareness, and motor responsiveness. Recovery rates slowed once the drug was discontinued, suggesting that amantadine accelerates recovery during active treatment but may not produce sustained benefits after withdrawal. This landmark trial provided the first Class I evidence for pharmacologic intervention in DoC and informed broad rehabilitation guidelines [47].

Smaller clinical trials have demonstrated positive results in both acute and chronic phases of recovery. A prospective pilot study by Tracy et al. followed 55 patients with severe TBI (GCS ≤ 8) during hospitalization, of whom 23 received amantadine. Treated patients showed greater short-term improvements in cognitive disability, as measured by changes in DRS scores, and were more likely to be discharged to specialized rehabilitation facilities [51]. In chronic TBI survivors, Kraus et al. showed that amantadine use was associated with improvements in executive function alongside increased prefrontal glucose metabolism on Fluorodeoxyglucose positron emission tomography (FDG-PET), providing biological support for its effects on arousal networks, even in chronic DoC [52]. This result aligns with other mechanistic studies of amantadine. In a population of post-anoxic brain injury DoC, Schnakers et al. demonstrated that amantadine treatment was associated with marked improvements in both motor and cognitive function and Coma Recovery Scale—Revised scores, with increased cortical metabolism observed by FDG-PET [53]. Although limited by sample size, these studies provide evidence that amantadine exerts restorative effects on higher-order cognition by modulating frontal networks.

While clinical evidence supports amantadine’s short-term efficacy, the durability of benefits after discontinuation remains uncertain. Nonetheless, its practical advantages, including favorable safety and tolerability, widespread availability, and relatively low cost, make it a pragmatic option for both acute inpatient care and long-term rehabilitation. Amantadine has the strongest evidence for use during the early subacute phase (4–16 weeks post-injury) in patients with DoCs and remains the only pharmacologic agent recommended by current guidelines for this indication [47,51,52,53].

### 5.2. Bromocriptine

Bromocriptine, a dopamine D2 receptor agonist, has been studied in TBI to modulate dopaminergic pathways in the striatum, prefrontal cortex, and mesolimbic system, which are critical for motor control, cognition, and motivation [54]. Beyond its DA effect, preclinical studies suggest that bromocriptine may reduce oxidative stress and lipid peroxidation, thereby helping preserve neuronal integrity [55]. It also influences prolactin release and circadian rhythms that contribute to metabolic homeostasis and mood regulation [56].

Clinical studies of bromocriptine have been mixed. In an early double-blind, placebo-controlled trial, McDowell et al. evaluated its effects in patients with moderate-to-severe TBI using a battery of neuropsychological tests, including dual-task paradigms, the Trail Making Test, Stroop Test, Verbal Fluency, and the Wisconsin Card Sorting Test [57]. Bromocriptine use was associated with selective improvements in executive function and attentional control; however, no benefit was observed for simpler working memory and sensorimotor tasks [57]. Subsequent studies have not replicated these findings. Whyte et al. conducted a pilot RCT in patients with moderate-to-severe TBI, with attention and processing speed as the primary outcomes [58]. The investigators found no significant differences between the bromocriptine and placebo groups, with several participants experiencing adverse effects, including agitation and nausea, raising concerns about drug tolerability and broader clinical use [58].

Bromocriptine may have a greater effect on motivation than on attention. Powell et al. examined patients with acquired brain injury using computer-based decision-making and reinforcement-learning models to measure initiation, persistence, and responsiveness to reward contingencies [59]. Those receiving bromocriptine showed greater willingness to initiate tasks and more adaptive, reinforcement-driven behavior than controls, suggesting that its dopaminergic effects in the mesolimbic and mesocortical pathways may help alleviate apathy and enhance goal-directed behavior following acquired brain injury [59].

Several case reports and case series also describe bromocriptine’s utility in managing paroxysmal sympathetic hyperactivity (PSH). PSH is a post-TBI syndrome of episodic autonomic dysregulation characterized by tachycardia, hypertension, hyperthermia, diaphoresis, and dystonic posturing. In one report, Russo et al. described a severe TBI patient with recurrent PSH episodes who showed a marked reduction in events after initiation of bromocriptine [60]. Baguley et al. reported decreased frequency and intensity of PSH episodes in a small series of patients prescribed bromocriptine, often in combination with other agents like morphine or propranolol [61]. These cases suggest that bromocriptine may act on hypothalamic and brainstem dopaminergic circuits to dampen sympathetic overactivity. Prevention of PSH is clinically relevant, as episodes are discomforting to patients and treatment allows for improved stability for rehabilitation.

Bromocriptine has also been evaluated as an adjunct to structured neurorehabilitation. Passler and Riggs reported on a case series of vegetative and minimally conscious TBI patients treated with bromocriptine, observing improvements in wakefulness, command-following, and therapy participation [62]. Clinical gains were most apparent when bromocriptine was combined with intensive rehabilitation, suggesting a possible synergistic effect. Given possible limitations from patient tolerability, bromocriptine might best be used in select cases under multidisciplinary guidance and structured rehabilitation. Bromocriptine has been studied in both acute and chronic TBI populations; however, evidence remains limited, and no specific recovery phase has demonstrated consistent benefit [57,58,59,62].

## 6. Acetylcholinesterase Inhibitors

### 6.1. Donepezil

Donepezil is a reversible AChEI that strengthens cholinergic neurotransmission. Additionally, it can upregulate nicotinic ACh receptors in cortical neurons, enhancing synaptic plasticity, attention, and learning. Donepezil is FDA-approved for the symptomatic treatment of Alzheimer’s disease and provides modest symptomatic improvements in cognition and activities of daily living, but it does not modify disease progression [63]. Preclinical studies suggest that donepezil may reduce neuroinflammatory activity, including microglial and astrocytic activation, although the clinical significance of these effects remains uncertain [63,64]. The rationale for evaluating donepezil in TBI comes from shared mechanisms. Both TBI and neurodegenerative disorders involve disruption of basal forebrain cholinergic pathways and inflammatory cascades that underlie long-term cognitive impairment. Donepezil, therefore, may be especially useful in the chronic phase of TBI, where persistent deficits in memory, attention, and executive function are common.

Several clinical studies support the use of donepezil for chronic post-TBI cognitive impairments. Zhang et al. conducted a 24-week randomized, placebo-controlled, crossover trial in 18 patients with moderate-to-severe TBI and persistent attention and memory deficits 2 to 24 months post-injury [65]. During the donepezil treatment phase, participants demonstrated significant improvements in sustained attention and short-term memory. Importantly, those who received donepezil first maintained many of their gains even after both washout and crossover to placebo, suggesting a carryover effect. Another clinical trial by Khateb et al. similarly showed that donepezil positively impacts attention, short-term memory, verbal memory, learning, executive function, and daily functioning [66]. Taken together, the evidence suggests a positive effect on broad aspects of memory and learning.

The utility of donepezil in earlier acute and subacute phases of TBI is less certain. In contrast to studies in the chronic phase, a retrospective analysis by Campbell et al. examined 55 patients with moderate-to-severe TBI who were prescribed donepezil during acute hospitalization compared to standard rehabilitation alone [67]. Investigators found no significant improvements in either cognitive or functional outcomes. These results suggest that the timing of administration may be a critical factor. While donepezil appears effective in addressing persistent cognitive deficits in the subacute and chronic phases, it may not confer additional benefit when introduced early. Taken together, these findings suggest that donepezil is most beneficial for persistent cognitive deficits during the subacute and chronic phases of recovery, whereas studies initiated during acute hospitalization have not demonstrated consistent benefit [65,66,67].

### 6.2. Rivastigmine

Rivastigmine is a dual cholinesterase inhibitor that acts on both AChE and butyrylcholinesterase (BuChE) through a pseudo-irreversible carbamate-binding mechanism [68]. In contrast to donepezil, which binds reversibly and dissociates relatively rapidly, rivastigmine produces prolonged enzyme inhibition that gradually reverses through spontaneous hydrolysis of the carbamylated enzyme complex [68]. This pharmacologic property provides sustained cholinergic enhancement without permanent enzyme inactivation [68]. While AChE is concentrated primarily within cortical and hippocampal synapses, BuChE is expressed more broadly in glial cells and peripheral tissues and appears to assume a greater role in cholinergic regulation under pathological conditions [68]. Because rivastigmine inhibits both AChE and BuChE, it may provide broader and more stable cholinergic modulation than agents selective for AChE alone [68].

In a 12-week RCT, Silver et al. evaluated rivastigmine for persistent cognitive symptoms following TBI [69]. Both treatment and placebo groups showed general improvement over time; however, post hoc analysis revealed that in participants with greater baseline memory impairment (≥25% deficit on the Hopkins Verbal Learning Test—Revised [HVLT-R]), rivastigmine was associated with greater gains in attention and verbal learning, reflected by improvements in the Cambridge Neuropsychological Test Automated Battery–Rapid Visual Information Processing (CANTAB RVIP) mean latency and HVLT-R total recall [70]. A 26-week open-label extension involving 127 of the original participants showed that this subgroup continued to improve through week 38 [70]. In contrast, a smaller crossover trial by Tenovuo et al. that enrolled less severely injured participants was not able to demonstrate objective improvements in cognition, concentration, and mood, with adverse effects limiting participation among some participants [71]. Rivastigmine may be most effective for individuals with moderate-to-severe memory deficits, although its benefits appear limited to specific cognitive domains.

The Rivastigmine for Veterans with Traumatic Brain Injury (RiVET) study expanded on these earlier trials by evaluating the transdermal patch formulation in veterans with moderate-to-severe post-traumatic memory impairment [72]. The patch was designed to achieve steadier plasma concentrations and minimize gastrointestinal side effects compared to oral dosing. Preliminary results indicated improvements in memory performance relative to baseline, accompanied by good tolerability and few adverse effects [72]. While these early findings are promising, published data remain limited, and a full peer-reviewed report of primary outcomes has yet to appear. Existing evidence is limited primarily to patients with chronic TBI and persistent memory impairment [70,71,72].

## 7. Selective Serotonin Reuptake Inhibitors

### 7.1. Sertraline

Sertraline is one of the most widely used SSRIs for post-traumatic depression, anxiety, and emotional dysregulation. It acts by inhibiting serotonin (5-HT) reuptake in the synaptic cleft, thereby enhancing serotonergic transmission. Sertraline also exerts modest effects on NE and DA reuptake [73]. Beyond its clinical indication for mood regulation, sertraline has neurorestorative properties relevant to post-TBI syndromes. It enhances neuroplasticity by upregulating brain-derived neurotrophic factor (BDNF) and activating the mitogen-activated protein (MAP) pathway, which together promote neuronal survival, dendritic growth, synaptic remodeling, and cortical reorganization after brain injury [73]. Additionally, MAP kinase activation leads to the phosphorylation of extracellular signal-regulated kinase 1 and 2 (ERK1/2) and the upregulation of B-cell lymphoma 2 (Bcl-2), a family of neuroprotective proteins that mitigates apoptotic signaling [74]. Regionally, sertraline’s mild dopaminergic activity in the nucleus accumbens and striatum enhances motivation, attention, and cognitive endurance, facilitating neurologic rehabilitation post-TBI.

A systematic review and meta-analysis by Reyes et al. evaluated the efficacy of sertraline for the treatment of depression and related neuropsychiatric symptoms following TBI. The review included four RCTs encompassing a total of 224 participants with post-TBI depression [75]. In their analysis, sertraline was safe and well-tolerated, but did not significantly improve depression severity, anxiety, or overall quality of life compared to placebo [75]. Gao et al. conducted a meta-analysis of five RCTs assessing the impact of sertraline on a broader range of post-TBI outcomes, including depression, anxiety, irritability, aggression, and quality of life. Similarly, their analysis found no conclusive benefit of sertraline across these domains [76]. Subgroup analyses revealed no differences in treatment response by injury severity, time since injury, or treatment duration. Despite its well-tolerated safety profile, the results from these two meta-analyses do not support the routine use of sertraline after TBI to improve neuropsychiatric or functional outcomes. Most studies have evaluated sertraline during the subacute and chronic phases following TBI. Routine use during acute hospitalization has not been established [75,76].

### 7.2. Fluoxetine

Fluoxetine, another widely available SSRI, enhances serotonergic signaling by inhibiting the reuptake of 5-HT. While this primary mechanism is shared with other SSRIs, fluoxetine exhibits broader neurobiological effects beyond 5-HT modulation. It has been shown to increase BDNF levels and activate its receptor, tropomyosin receptor kinase B (TrkB), even in the absence of 5-HT transporter activity [77]. This pathway supports neuronal survival and synaptic remodeling. In parallel, fluoxetine influences cellular energy metabolism by enhancing oxidative phosphorylation and upregulating D1-like dopamine receptor activity, thereby promoting motivation, alertness, and cognitive endurance [78]. It also engages the Akt1 (protein kinase B alpha) signaling cascade, a pathway associated with neurogenesis and protection against secondary neuronal injury [78]. In a murine controlled cortical impact (CCI) model, Wang et al. demonstrated that chronic fluoxetine exposure enhances hippocampal neurogenesis and induces epigenetic changes that promote histone H3 acetylation and the expression of methyl-CpG-binding proteins, linked to heightened BDNF signaling and synaptic plasticity [79]. In an additional CCI model of severe TBI, fluoxetine improved motor function and coordination on the rota-rod test while reducing lung and intestinal injury secondary to systemic inflammation after TBI [79].

Clinical evidence remains scarce and preliminary. Sloan et al. conducted an open-label case series examining fluoxetine for emotional lability in patients with acquired brain injury, including one individual with TBI [80]. Using a modified Lawson and MacLeod rating scale, the authors observed a notable reduction in mood instability and emotional outbursts within a week of treatment, with no significant side effects reported [80]. In a separate case series, Horsfield et al. evaluated long-term fluoxetine administration (20–60 mg/day for 8 months) in 5 patients with TBI to assess its impact on mood and cognition [81]. Improvements were noted in depressive symptoms and in select neuropsychological measures, including attention and working memory, as assessed by the Hamilton Depression Rating Scale, the Trail Making Test A, and the WAIS-III Letter–Number Sequencing [81]. While these small, uncontrolled studies suggest potential neuropsychiatric and cognitive benefits, their limited sample sizes and methodological constraints highlight the need for larger RCTs to determine the therapeutic value of fluoxetine in TBI recovery. Clinical evidence is currently limited to patients in the chronic phase of recovery, and additional studies are needed to define its role earlier after injury [80,81].

## 8. Tolerability of Pharmacologic Neuromodulators

Across the drug classes described, the tolerability and efficacy profiles vary substantially. Amantadine is typically well tolerated but can occasionally cause insomnia, agitation, or hallucinations, particularly at higher doses or in patients with pre-existing psychiatric disorders [47]. Methylphenidate carries typical stimulant-related risks such as mild increases in heart rate and blood pressure and, with long-term use, a potential risk of tolerance or dependence; however, these are uncommon under appropriate clinical supervision [26]. Modafinil has a generally favorable side-effect profile, with headache, nausea, and mild anxiety being the most frequently reported side effects; serious allergic reactions are uncommon. Bromocriptine is less tolerated with chronic use, being associated with nausea, orthostatic hypotension, and mood changes that limit its routine prescription [57]. Cholinesterase inhibitors like donepezil and rivastigmine are generally well tolerated, though gastrointestinal upset can occur [65]. In the acute post-TBI phase, mild side effects may be more tolerated if there is potential to improve a DoC with a depressed level of consciousness. Symptoms of intolerability may not even manifest in those more severely injured, including those in a coma or minimally conscious state. In the chronic phase of recovery, few studies have examined outcomes beyond a few months; therefore, the long-term safety and tolerability of these medications remain uncertain.

## 9. Non-Pharmacologic Neuromodulation

Non-pharmacological neuromodulation has gained attention as a potential adjunct to conventional rehabilitation in patients with TBI, particularly for symptoms that are resistant to pharmacologic treatment alone. Non-invasive brain stimulation (NIBS) techniques, including rTMS and tDCS, are appealing because they avoid surgical risk, are generally well tolerated, and can be delivered in both inpatient and outpatient rehabilitation settings. These approaches aim to modulate cortical excitability and network connectivity in regions commonly disrupted after TBI. Table 2 summarizes the major non-pharmacologic neuromodulation techniques, including their proposed mechanisms, principal clinical indications, current strength of evidence, and important limitations.

### 9.1. Repetitive Transcranial Magnetic Stimulation

Among NIBS modalities, rTMS is the most extensively studied in TBI. By delivering focal magnetic pulses to targeted cortical areas, rTMS has been primarily investigated for post-TBI depression, anxiety, headache, and cognitive dysfunction. A meta-analysis by Tsai et al. found that stimulation of the left dorsolateral prefrontal cortex (DLPFC) was associated with short-term antidepressant effects, though benefits often diminished over time [8]. The left DLPFC is typically hypoactive in depression and plays a key role in cognition and regulation of limbic emotional circuits [8]. Excitatory stimulation is thought to enhance prefrontal activity and the regulation of limbic regions, and to induce neuroplastic changes, including increased neurotrophic signaling. Ongoing or maintenance stimulation may be necessary to sustain clinical response [8].

The CAPTAIN-rTMS trial represents an important advance in the study of neuromodulation for TBI rehabilitation [9]. In this three-arm RCT, Verisezan Roșu et al. compared rTMS alone, Cerebrolysin alone, and their combination in patients with TBI [10]. Targeting the DLPFC, rTMS was designed to modulate cognitive networks through activity-dependent plasticity, while Cerebrolysin was included as a comparator based on proposed neurotrophic and neuroprotective properties described in preclinical studies; however, these mechanisms were not evaluated in the clinical trial. The combined treatment arm demonstrated greater improvements in cognitive and functional outcomes than either intervention alone, suggesting a synergistic effect between interventions [10]. Although limited by modest sample size and short follow-up, the trial provides the first RCT evidence supporting the feasibility and biological plausibility of combining pharmacologic and non-pharmacologic neuromodulation strategies in TBI.

A secondary analysis of the CAPTAIN-rTMS cohort by Olivia et al. used quantitative electroencephalography (EEG) to assess neurophysiological effects of treatment [9]. Pre- and post-rTMS EEG recordings were analyzed using spectral power and functional connectivity measures to characterize changes in cortical oscillatory activity. The authors observed changes in cortical rhythms and connectivity patterns consistent with network-level plasticity and target engagement [9]. While these findings provide mechanistic evidence of rTMS-induced neurophysiologic change, durable cognitive and functional outcomes were not directly examined. Most clinical trials have enrolled patients during the subacute or chronic rehabilitation phase, and evidence supporting acute application remains limited [8,9,10].

### 9.2. Transcranial Direct Current Stimulation

By delivering low-intensity electrical current through scalp electrodes, tDCS modulates cortical excitability and learning-related plasticity in a state-dependent manner rather than directly eliciting neuronal firing. Clinical evidence to date comes largely from small pilot studies in patients with mild-to-moderate or chronic TBI, in which tDCS has been paired with concurrent cognitive or motor training.

In an often-cited pilot study in mild-to-moderate TBI, Quinn et al. demonstrated that pairing prefrontal tDCS with structured memory training yielded greater improvements in working memory performance than cognitive training alone [11]. Neuroimaging analyses further showed increased functional connectivity between prefrontal and insular regions following treatment, suggesting enhanced integration within cognitive control and salience networks. Similarly, Ulam et al. conducted a randomized, sham-controlled crossover study in patients with chronic TBI, applying anodal tDCS to the left DLPFC during cognitive task performance [12]. Active stimulation was associated with modest improvements in attention and executive function compared with sham conditions. Beyond cognition, Kang et al. showed that motor cortex tDCS, paired with physical therapy, led to greater improvements in motor performance than physical therapy alone in TBI patients with motor deficits [13]. Collectively, these studies suggest that tDCS may enhance task-dependent plasticity and network reorganization across both cognitive and motor domains. However, small sample sizes, heterogeneous protocols, and limited follow-up constrain conclusions regarding durability and generalizability. Current evidence is derived primarily from chronic TBI populations undergoing concurrent rehabilitation therapies [11,12,13].

### 9.3. Electroconvulsive Therapy

Electroconvulsive therapy (ECT) is a noninvasive neuromodulatory intervention that delivers controlled high electrical currents through scalp electrodes to induce a brief, generalized seizure under anesthesia. This process is thought to produce widespread neurochemical and network-level changes, including modulation of frontolimbic circuits and upregulation of neurotrophic signaling, resulting in improvement in severe treatment-resistant psychiatric conditions such as major depression, catatonia, and psychosis. Early foundational work by Max Fink established that the presence of structural brain disease, including prior TBI, is not an absolute contraindication to ECT [82]. Subsequent advances in technique and safety were defined by studies by Charles H. Kellner et al., which demonstrated that modern ECT paradigms—particularly right unilateral electrode placement and brief- or ultra-brief-pulse stimulation—substantially reduce cognitive adverse effects while maintaining antidepressant efficacy [83]. Although not specific to TBI, these studies are frequently cited in the TBI literature as the methodological basis for safer application in patients with pre-existing cognitive vulnerability.

Complementary clinical evidence from case series by Andrew F. Krystal et al. further supported ECT use in patients with neurologic injury, describing meaningful improvement in treatment-resistant depression and catatonia without evidence of permanent cognitive decline [84]. More recent case series have specifically examined ECT outcomes in individuals with prior TBI and refractory psychiatric symptoms. Kant et al. reported cognitive and mood improvements following ECT in patients with a history of closed-head injury [85]. Similarly, Tang et al. demonstrated improvement in post-traumatic stress disorder (PTSD) symptoms with comorbid depression [86]. Collectively, the available evidence suggests the safety of ECT as a treatment in severe, treatment-resistant psychiatric sequelae of TBI; however, high cost, limited availability, and scarce long-term data limit its use. ECT is generally reserved for chronic treatment-refractory psychiatric sequelae following TBI rather than for acute neurological recovery [82,83,84,85,86].

### 9.4. Vagus Nerve Stimulation

VNS has been explored as a neuromodulatory strategy in TBI because of its capacity to influence arousal, attention, inflammation, and neuroplasticity through modulation of deep nuclei—particularly the locus coeruleus (noradrenergic) and basal forebrain (cholinergic)—as well as through activation of the cholinergic anti-inflammatory reflex [87]. Enhancing these systems can mitigate secondary injury processes, including cerebral edema and blood–brain barrier disruption, while priming neural circuits for rehabilitation-driven learning. In a feasibility and safety study, Hakon et al. evaluated transcutaneous VNS in a small cohort of patients with severe TBI, including those with diffuse axonal injury, approximately one month after injury [88]. The intervention was well tolerated, with no serious stimulation-related adverse events; however, the study was not powered to assess efficacy.

Proof-of-principle evidence was provided by Corazzol et al., who reported on a patient with a chronic DoC following TBI treated with an implanted VNS [89]. Using longitudinal behavioral assessments alongside EEG and PET, the authors demonstrated improvements in arousal and responsiveness accompanied by increased cortical activity and changes in large-scale network connectivity, particularly within frontoparietal and thalamocortical systems implicated in consciousness. Although limited to a single case, this study is frequently cited as a demonstration that VNS can modulate arousal-related networks in severe acquired brain injury. Currently, VNS use for TBI sequelae remains investigational. Evidence remains investigational, with studies spanning both subacute and chronic TBI populations [88,89].

### 9.5. Deep Brain Stimulation

DBS has been explored in chronic moderate-to-severe TBI to restore function in underactive thalamocortical arousal and cognitive-control circuits, often targeting the central thalamus; however, data remain limited. In a single-patient, proof-of-concept, double-blind crossover study by Schiff et al., bilateral central thalamic DBS was delivered to a patient who remained in a minimally conscious state for years after severe TBI [90]. Stimulation produced measurable improvements in responsiveness and goal-directed behaviors across on/off conditions, providing early causal evidence that thalamic stimulation can engage large-scale networks supporting consciousness and behavior after TBI. A more recent phase 1 randomized feasibility study by the same group evaluated chronic central lateral/dorsal tegmental tract-targeted thalamic DBS in patients with chronic moderate-to-severe TBI [91]. All participants were successfully implanted, most completed the protocol, and the primary cognitive endpoint, executive control indexed by processing speed on the Trail Making Test Part B, showed improvements of ~15% to 52% from baseline, supporting both target engagement and a signal of potential benefit in higher-order cognition. While still early-stage, such studies establish plausibility and equipoise for further study. Given its invasiveness, DBS is likely limited to select cases with high potential for cognitive recovery. Published experience has almost exclusively involved patients with chronic DoCs or cognitive impairment following severe TBI [90,91].

Table 3 and Table 4 summarize comparison and evidence of non-pharmacologic neuromodulation techniques in traumatic brain Injury recovery.

## 10. Current Guidelines & Clinical Approach

### 10.1. Limitations in the Current Literature

The quality of evidence supporting the use of neuromodulators after TBI generally remains low to moderate, often relying on non-controlled studies. A major limitation of the current RCT literature is that many studies are underpowered due to small sample sizes. Interpretation is further complicated by patient-level heterogeneity across trial cohorts, as studies vary widely in injury severity, time since injury, and recovery phase. Furthermore, because interventions have been initiated at different stages of recovery (acute, subacute, and chronic), identifying the optimal therapeutic window for individual neuromodulatory interventions remains difficult. In addition, outcome measures differ considerably between studies, with some trials emphasizing global functional scales such as the Disability Rating Scale. In contrast, others rely on domain-specific cognitive tests or mood and quality-of-life measures, limiting cross-study comparison and synthesis. Short treatment durations and limited follow-up weaken conclusions, as benefits observed during active treatment, such as the accelerated recovery seen with amantadine, often diminish after discontinuation, and other neuromodulator trials provide little insight into long-term efficacy or safety. Finally, the near absence of head-to-head RCTs leaves questions of relative efficacy, optimal sequencing, and combination therapy largely unresolved and reliant on indirect comparisons and clinical judgment.

### 10.2. Guidelines

Guideline-based recommendations for neuromodulators in TBI are limited, with only a small number of agents supported by low or moderate-quality evidence. The most robust recommendations from the American Academy of Neurology (AAN) and the American Congress of Rehabilitation Medicine (ACRM) endorse amantadine for severe TBI with DoC during the subacute period (approximately 4–16 weeks post-injury) to accelerate functional recovery [92]. Outside of DoC, neuromodulation is symptom-targeted and guided by expert opinion. Methylphenidate use is incorporated into rehabilitation practice frameworks, including International Cognitive Rehabilitation Guidelines for Traumatic Brain Injury (INCOG) recommendations to improve attention, processing speed, and executive function [93]. Current guidelines do not support the routine use of other agents. Importantly, medications are not recommended as standalone therapies. The ACRM, AAN, and INCOG emphasize that early, intensive, and task-specific neurorehabilitation—including physical, occupational, speech-language, and cognitive therapy—is recommended across all injury severities, with strong evidence that early inpatient rehabilitation improves long-term functional outcomes in moderate-to-severe TBI [92,93]. Noninvasive neuromodulation techniques such as rTMS and tDCS are increasingly studied as adjuncts but remain investigational in current guidelines.

### 10.3. Clinical Approach

**Figure 1:**
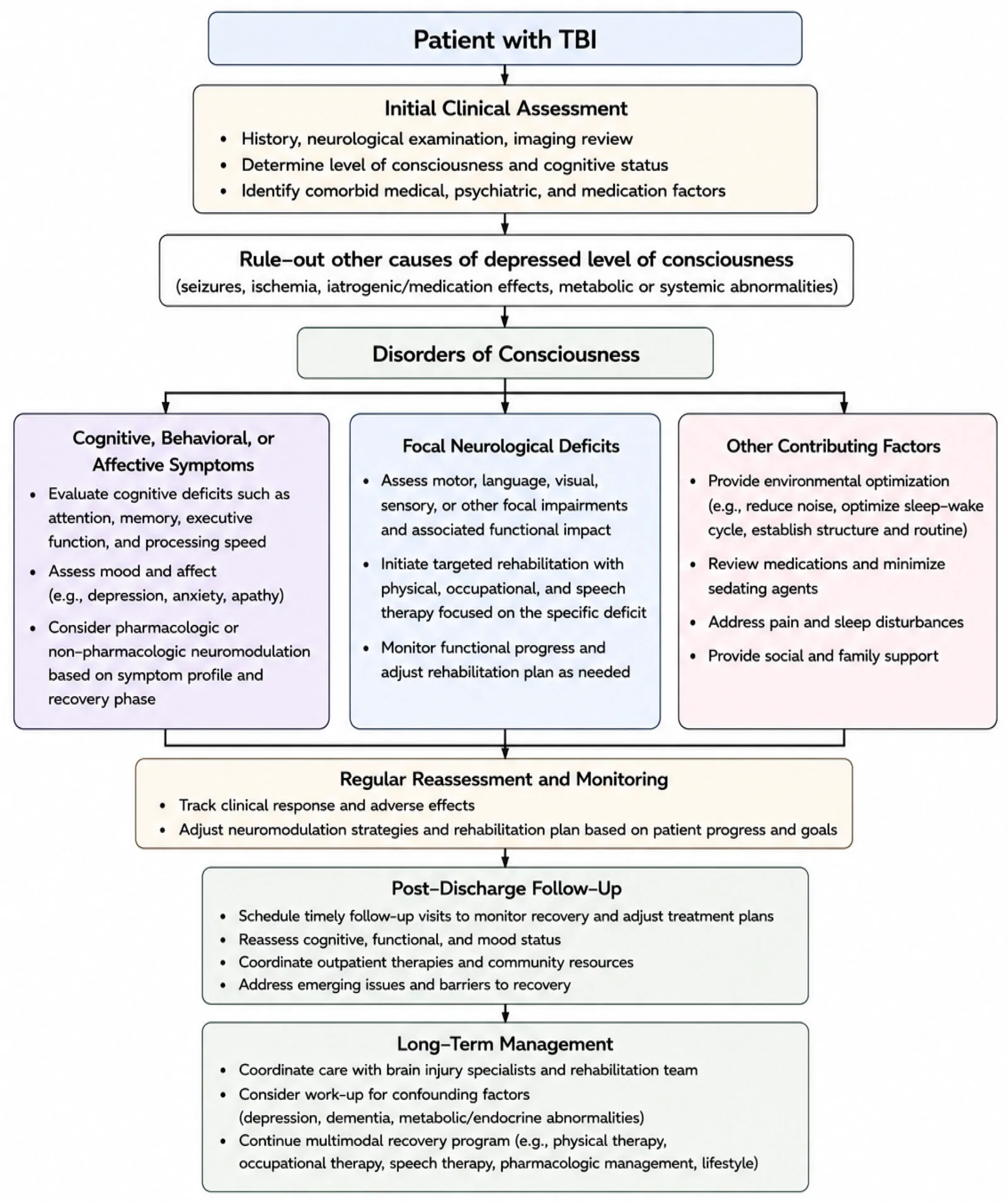
Proposed clinical approach to neuromodulation following traumatic brain injury (TBI). This algorithm represents the authors’ synthesis of the current literature and available guideline recommendations, including the American Academy of Neurology (AAN) practice guideline for disorders of consciousness (DoC) and the INCOG 2.0 cognitive rehabilitation guidelines. It is intended to provide a practical framework for clinical decision-making rather than a formal clinical practice guideline. Treatment selection should be individualized based on injury severity, recovery phase, patient-specific goals, and institutional practice. Developed by the authors from a synthesis of the current literature and guideline recommendations, including Refs. [92,93].

In the absence of widely accepted guidelines, neuromodulator use remains largely dictated by expert opinion and clinical judgment (Figure 1). Pharmacologic neuromodulatory therapies should be considered within a stepwise, individualized framework: first, excluding reversible contributors to impaired consciousness or cognition (e.g., seizures, medication effects, metabolic or endocrine disturbances); then, selecting agents based on several factors, including injury severity, recovery phase, and symptom profile, with close longitudinal monitoring.

Therapeutic response to neuromodulators likely varies with the phase of recovery and the injury phenotype. In the acute post-TBI phase, amantadine shows the strongest efficacy data in severe TBI with DoCs and depressed levels of consciousness [47]. This is clinically important, as overt consciousness or improved wakefulness can profoundly impact clinical decision-making, discussions about goals of care, and rehabilitation potential. Additionally, it is ideal to initiate treatment in the early recovery phase when neuroplastic changes are most active [47]. Based on the available literature, a trial of amantadine is reasonable in appropriately selected patients with DoCs when the potential benefits outweigh treatment risks.

In the chronic care of TBI, neuromodulators are symptom-targeted. Methylphenidate is considered for patients in rehabilitation who experience persistent inattention, slowed thinking, or executive dysfunction, as it can improve processing speed and motivation [26]. Modafinil may be most beneficial for those with mild-to-moderate injuries who experience fatigue or excessive sleepiness that interferes with therapy participation [42]. Given their extensive use and evidence in Alzheimer’s disease and other dementias, AChE inhibitors donepezil and rivastigmine may be considered for chronic-phase TBI with persistent memory and attention problems, particularly when cholinergic signaling deficits or concurrent dementias are suspected [65]. The SSRI fluoxetine may have added value when mood symptoms, irritability, or emotional lability accompany cognitive challenges. Lastly, sertraline remains one of the most frequently prescribed SSRIs for managing post-traumatic depression, anxiety, and emotional dysregulation; however, evidence regarding its efficacy in improving neuropsychiatric or cognitive outcomes after TBI has been mixed [75]. A multimodal recovery program should be implemented that includes multidisciplinary participation from rehabilitation therapy, medical providers, mental health providers, and lifestyle modification. For patients with treatment-refractory symptoms, clinicians should reassess for confounding factors, including depression, dementia, and metabolic and endocrine dysfunctions. Non-pharmacologic neuromodulator therapies remain investigational and should only be offered as part of clinical trials.

## 11. Conclusions

There is emerging evidence for both pharmacologic and non-pharmacologic neuromodulation to improve outcomes after TBI. Pharmacologic neuromodulatory therapies may be useful in the acute and subacute phase for DoCs, while in the chronic phase, neuromodulators can be used in a symptom-targeted manner. Rather than relying on any single therapy, a multimodal approach incorporating both pharmacologic and non-pharmacologic therapies, individualized to disease severity, phase of recovery, and patient-specific symptoms, may represent the most promising strategy for optimizing neurological recovery. High-quality research is needed, with priority given to larger efficacy trials, longitudinal studies, and comparative analyses of various therapies. Future work should identify clinical, neuroimaging, electrophysiologic, and biomarker predictors of treatment response to specific neuromodulatory therapies, enabling more individualized and precision-based rehabilitation strategies. As evidence from rigorous randomized trials and long-term follow-up continues to grow, neuromodulation is poised to become an increasingly used, effective, and durable component of recovery-oriented care for patients with TBI.

## Figures and Tables

**Table 1:** Neuromodulation and Cognitive-Enhancing Pharmacotherapies Used in Traumatic Brain Injury Recovery: Mechanisms, Dosing, Clinical Applications, and Adverse Effects.

Drug Class	Agent	Primary Mechanism(s)	Typical Dose Range	Main Clinical Uses for TBI	Recovery Phase with Strongest Clinical Evidence	Common Side Effects
CNS Stimulant	Methylphenidate	Inhibits DAT and NET, increasing dopamine and norepinephrine availability; enhances activity within prefrontal, striatal, and nucleus accumbens networks.	5–10 mg twice daily (max ~60 mg/day)	Improves attention, processing speed, and executive function Reduces apathy and impulsivity May provide sustained cognitive and emotional benefits in selected patients	Subacute–Chronic	Hypertension Tachycardia Anxiety Irritability Insomnia
	Modafinil	Inhibits DAT and modulates serotonin, norepinephrine, histamine, hypocretin, glutamate, and GABA pathways to promote cortical arousal.	100–200 mg every morning (up to 400 mg/day)	Promotes wakefulness Reduces excessive daytime sleepiness and fatigue May improve attention and participation in rehabilitation	Subacute–Chronic (fatigue/EDS)	Headache Anxiety Insomnia Decreased appetite
Dopaminergic Agent	Amantadine	NMDA receptor antagonist that enhances dopaminergic signaling through delayed dopamine reuptake and increased postsynaptic receptor expression.	100–200 mg twice daily	Improves arousal and awareness in severe TBI Accelerates recovery in disorders of consciousness May improve executive function Strongest evidence during early/subacute recovery	Early subacute (4–16 weeks post-injury)	Confusion Hallucinations Insomnia Orthostatic hypotension
	Bromocriptine	Dopamine D2 receptor agonist that modulates striatal and prefrontal circuits and preclinical studies suggest it may reduce oxidative stress.	2.5–5 mg two or three times daily (start low and titrate cautiously)	May improve arousal in severe TBI May enhance motivation, attention, and motor function Occasionally used for apathy or paroxysmal sympathetic hyperactivity Evidence remains limited	No clearly established optimal phase	Nausea Orthostatic hypotension Hallucinations Impulse-control disorders
AChE Inhibitor	Donepezil	Reversible acetylcholinesterase inhibitor that increases synaptic acetylcholine concentrations and preclinical studies suggest reduced neuroinflammation.	5–10 mg daily	Improves attention and memory in chronic or subacute TBI Limited utility during the acute phase	Subacute–Chronic	Nausea Diarrhea Bradycardia Insomnia
	Rivastigmine	Pseudo-irreversible inhibitor of both acetylcholinesterase and butyrylcholinesterase, producing sustained cholinergic enhancement.	1.5–6 mg twice daily (oral) or 4.6–9.5 mg/24 h patch	May provide modest cognitive benefits in moderate-to-severe chronic TBI Generally well tolerated	Chronic	Nausea Vomiting Dizziness Weight loss
SSRI	Sertraline	Inhibits serotonin reuptake and may enhance BDNF, MAPK/ERK, and Bcl-2 signaling pathways.	25–200 mg daily	Commonly used for post-TBI depression and anxiety No consistent evidence for cognitive improvement	Subacute–Chronic	Nausea Sexual dysfunction Insomnia Headache
	Fluoxetine	Inhibits serotonin reuptake and modulates BDNF/TrkB, dopaminergic signaling, oxidative metabolism, and Akt1 pathways.	10–80 mg daily	May improve mood and emotional regulation after TBI Cognitive benefits remain uncertain	Chronic	Nausea Anxiety Insomnia Sexual dysfunction

**Table 2:** Evidence Summary of Pharmacologic Neuromodulators in Traumatic Brain Injury Recovery.

Drug Class	Agent	FDA Approved Indication	TBI Indication	Highest Level of Clinical Evidence	Approximate RCTs	Overall Evidence	Representative References
CNS Stimulant	Methylphenidate	ADHD, narcolepsy	Off-label	Multiple RCTs	~6	Low–moderate	Whyte et al. [26]; Peattie et al. [34]; Barnett & Reid [35]; van der Veen et al. [36]
	Modafinil	Narcolepsy	Off-label	Small RCTs	2–3	Low	Kaiser et al. [42]; Dhamapurkar et al. [43]; Seifi et al. [44]
Dopaminergic Agent	Amantadine	Parkinson disease	Off-label	AAN guideline + Class I RCT	3–5	Moderate	Giacino et al. [47]; Tracy et al. [51]; Kraus et al. [52]
	Bromocriptine	Parkinson disease	Off-label	Pilot RCTs	2	Very low	McDowell et al. [57]; Whyte et al. [58]; Powell et al. [59]
AChE Inhibitor	Donepezil	Alzheimer’s disease	Off-label	Small RCTs	2	Low	Zhang et al. [65]; Khateb et al. [66]
	Rivastigmine	Alzheimer’s disease	Off-label	Small RCTs	2	Low	Silver et al. [69,70]; Tenovuo et al. [71]; RiVET [72]
SSRI	Sertraline	Major depressive disorder	Off-label	Meta-analysis	4–5	Low	Reyes et al. [75]; Gao et al. [76]
	Fluoxetine	Major depressive disorder	Off-label	Case series	0	Very low	Sloan et al. [80]; Horsfield et al. [81]

**Table 3:** Comparison of Non-Pharmacologic Neuromodulation Techniques for Traumatic Brain Injury Recovery [8,9,10,11,12,13,82,83,84,85,86,87,88,89,90,91].

Intervention	Primary Mechanism	Recovery Phase with Strongest Clinical Evidence	Primary Clinical Indications for TBI	Common Side Effects
Repetitive Transcranial Magnetic Stimulation (rTMS)	Focal magnetic stimulation increases cortical excitability, modulates frontolimbic and thalamocortical networks, and promotes activity-dependent neuroplasticity	Subacute–Chronic	Depression, cognitive dysfunction, executive dysfunction, headache	Scalp discomfort, headache, facial muscle twitching, rare seizure
Transcranial Direct Current Stimulation (tDCS)	Low-intensity electrical current modulates cortical excitability and facilitates learning-dependent plasticity	Chronic	Attention, working memory, executive dysfunction, motor rehabilitation	Mild scalp tingling, itching, skin erythema, headache
Electroconvulsive Therapy (ECT)	Induces generalized seizure with widespread neurochemical and network modulation, increasing neuroplasticity and neurotrophic signaling	Chronic	Treatment-resistant depression, catatonia, and severe psychiatric sequelae	Transient confusion, headache, myalgias, short-term memory impairment, anesthesia-related risks
Vagus Nerve Stimulation (VNS)	Activates vagal afferents to modulate locus coeruleus and basal forebrain pathways while reducing neuroinflammation and enhancing neuroplasticity	Subacute–Chronic	Disorders of consciousness, arousal, investigational disorders of consciousness and cognitive recovery	Hoarseness, cough, throat discomfort, dysphagia (implantable); mild skin irritation (transcutaneous)
Deep Brain Stimulation (DBS)	Direct electrical stimulation of thalamocortical arousal networks to restore large-scale network activity	Chronic	Chronic disorders of consciousness and severe cognitive impairment	Intracranial hemorrhage, infection, lead migration, hardware malfunction, stimulation-induced mood or cognitive changes

**Table 4:** Evidence of Non-Pharmacologic Neuromodulation Techniques in Traumatic Brain Injury Recovery.

Intervention	FDA-Approved Indication	TBI Indication	Strength of Evidence	Major Limitations	Representative Reference
Repetitive Transcranial Magnetic Stimulation (rTMS)	Depression, OCD	Off-label	Moderate (multiple RCTs and meta-analyses)	Small studies, heterogeneous protocols, uncertain long-term durability	Tsai et al. [8], CAPTAIN-rTMS [9]
Transcranial Direct Current Stimulation (tDCS)	None	Investigational	Low–moderate (small RCTs/pilot studies)	Variable stimulation parameters; typically requires concurrent rehabilitation; limited follow-up	Quinn et al. [11], Ulam et al. [12]
Electroconvulsive Therapy (ECT)	Depression	Psychiatric sequelae	Low (case series and observational studies)	Limited TBI-specific evidence; anesthesia required; reserved for selected psychiatric indications	Kant et al. [85]
Vagus Nerve Stimulation (VNS)	Epilepsy, Depression	Investigational	Very Low (pilot studies and case reports)	Mostly investigational; invasive implantation for conventional VNS; limited efficacy data	Hakon et al. [88]; Corazzol et al. [89]
Deep Brain Stimulation (DBS)	Parkinson disease	Investigational	Very Low (proof-of-concept and feasibility studies)	Highly invasive; highly selected patients; limited availability and clinical experience	Schiff et al. [90,91]

## Data Availability

No datasets were generated or analyzed during the current study.
